# Electrokinetic Properties of TiO_2_ Nanotubular Surfaces

**DOI:** 10.1186/s11671-016-1594-3

**Published:** 2016-08-25

**Authors:** Martina Lorenzetti, Ekaterina Gongadze, Mukta Kulkarni, Ita Junkar, Aleš Iglič

**Affiliations:** 1 Jožef Stefan Institute, Jamova cesta 39, 1000 Ljubljana, Slovenia; 2 Faculty of Electrical Engineering, University of Ljubljana, 1000 Ljubljana, Slovenia

**Keywords:** TiO_2_ nanostructured surfaces, Zeta potential, Surface charge, TiO_2_ nanotubes, Anodization

## Abstract

Surface charge is one of the most significant properties for the characterisation of a biomaterial, being a key parameter in the interaction of the body implant with the surrounding living tissues. The present study concerns the systematic assessment of the surface charge of electrochemically anodized TiO_2_ nanotubular surfaces, proposed as coating material for Ti body implants. Biologically relevant electrolytes (NaCl, PBS, cell medium) were chosen to simulate the physiological conditions. The measurements were accomplished as titration curves at low electrolytic concentration (10^−3^ M) and as single points at fixed pH but at various electrolytic concentrations (up to 0.1 M). The results showed that all the surfaces were negatively charged at physiological pH. However, the zeta potential values were dependent on the electrolytic conditions (electrolyte ion concentration, multivalence of the electrolyte ions, etc.) and on the surface characteristics (nanotubes top diameter, average porosity, exposed surface area, wettability, affinity to specific ions, etc.). Accordingly, various explanations were proposed to support the different experimental data among the surfaces. Theoretical model of electric double layer which takes into account the asymmetric finite size of ions in electrolyte and orientational ordering of water dipoles was modified according to our specific system in order to interpret the experimental data. Experimental results were in agreement with the theoretical predictions. Overall, our results contribute to enrich the state-of-art on the characterisation of nanostructured implant surfaces at the bio-interface, especially in case of topographically porous and rough surfaces.

## Background

Titanium and its alloys are generally considered suitable materials for metallic implants [1, 2], even though they lack per se in terms of biocompatibility, due to their intrinsic inertness. One of the strategies to overcome this issue is to improve their surface properties (i.e. surface roughness and topography, chemical composition, wettability, charge) by creating nanostructured surfaces able to ensure a beneficial, pre-conditioning film of ions and proteins, which will support the further cell adhesion and wound healing, have antibacterial properties, etc.

The surface modifications of Ti-based implants nano-topography have been extensively studied by several authors [3, 4]. In particular, the use of self-assembled titanium dioxide (TiO_2_) nanostructures have found much interest in various fields of functional biomedical applications, especially attributable to the great advantage of gaining a very highly exposed (effective) surface area. In fact, the extremely high effective surface area of vertically oriented TiO_2_ nanotubes (NTs) results in a superior surface energy, leading to an increase in the initial protein adsorption that can enhance cellular interactions (increased osseointegration, antibacterial activity, mitigation of the inflammatory response, etc.) [3, 5–8]. Recently, research and development in the area of synthesis and applications of different nanostructured TiO_2_ materials has been greatly increasing. The TiO_2_ nanostructures are primarily categorised by their different methods of preparation, such as sol-gel, hydrothermal treatment, assisted-template synthesis, and electrochemical anodization (EA) [9]. EA of Ti is the preferred method for growing self-assembled TiO_2_ nanotubular structures directly on the substrate materials, as it enables a good control over their geometry and specific diameters, long-range order as well as an ease of application [9, 10]. In view of that, herein we obtained nanotubes with specific diameters by optimising experimental parameters during electrochemical anodization. Such structures were shown to be highly valuable for biological applications not only from the point of increased surface area and possibility to be used as reservoirs for medications, but also for the selective attachment of proteins, which further dictate adhesion of cells. It has been previously shown [11] that binding of proteins is highly influenced by the size of the nanotube diameter, which is due not only to the increased surface area but also to the possibility of proteins to enter into the narrow interior of the nanotubes (only smaller proteins can enter the NTs 15 nm diameter interior). Thus, different nanotube diameters could play an important role in the designing of implantable devices, as it is possible to selectively promote the growth of one cell type over another.

Together with the choice for the most suitable surface modification technique, the evaluation of the physico-chemical properties of a biomaterial surface is compulsory for the proper design of body implants and to predict a priori the influence of the material properties on the events occurring at the bio-interface [12, 13]. In fact, the surface properties are responsible for the interactions with the surrounding tissues and, influencing the “race for the surface” between cells and bacteria [14], affect the consequent acceptance of the implant. The most studied surface characteristics concern the roughness and topography, chemical composition, and wettability, either at the micro- or nano-scale. Besides, the assessment of the surface charge of solids becomes fundamental when the adsorption of certain ions and proteins from the body fluids to the implant is considered. Therefore, the study of the implant surface electrokinetic behaviour is highly valuable, especially in connection with surface topography and wettability.

In fact, the electrostatic interactions are fundamental for binding with ions, plasma proteins and, subsequently, cells and tissues. For instance, the local surface charge can be related to the surface nano-roughness provided by the presence of TiO_2_ NTs. In particular, the high surface charge density at the sharp or convex edges of the nanotubes provides prominent binding sites for mono/divalent ions and for proteins with a distinctive quadrupolar charge distribution, able to mediate the further adhesion of osteoblasts [15]. Accordingly, in our previous study, the binding of certain plasma proteins (important in wound healing and inflammatory responses) to TiO_2_ NTs with different diameters was studied [11]. In that case, the surface charge of the nanotubes was assessed by measuring the zeta potential of NTs sheared out of the Ti-substrate. Even though the used electrophoretic mobility technique allowed the determination of *ζ* of the NTs in suspension, no significant difference was observed between the NTs with different diameters, either in terms of isoelectric point (IEP) values or *ζ* magnitude in dependence of the pH. Therefore, in the next step, the zeta potential of self-assembled TiO_2_ NTs on Ti surfaces was measured by streaming potential technique, when still adhered to the substrate [16]. Streaming potential represents a reliable method to evaluate the surface charge by determining the zeta potential (*ζ*) at the interface between the material and the fluid around [12, 13, 16]. Differently from electrophoresis and electroacoustic techniques, the streaming potential technique consists in the formation of an electric field as the electrolyte flows tangentially to a stationary charged solid surface, so that the zeta potential is calculated out of the generated streaming potential.

Thus, the purpose of this work was to assess measurements of surface charge of electrochemically anodized titanium-based substrates used for hard tissue replacement. Zeta potential of TiO_2_ nanotubes and various elongated titanate derivatives has been already assessed via electrophoretic mobility measurements [17–19]. However, this technique does not allow the direct determination of surface charge on solid materials, only on suspensions or dispersions.

To our best knowledge, for the first time an electrokinetic study of zeta potential of TiO_2_ nanotube-coated titanium was accomplished systematically in biologically relevant electrolytes. The experimental data were supported by theoretical models, which resulted in equations specific to our system but applicable also to other nanoporous structures.

## Methods

### Growth of TiO_2_ Nanotubes

The TiO_2_ nanostructures were fabricated according to the electrochemical anodization method published earlier [9–11, 16, 20, 21]. Briefly, Ti foils of 0.1 mm thickness (99.6 % purity, Advent Research Materials, England) were used as starting material for the fabrication of TiO_2_ nanotubes. Prior to anodization, Ti foils were cleaned by successive ultrasonication in acetone, ethanol, and deionised water for 5 min each and dried under nitrogen stream. Chemicals required for anodization, such as hydrofluoric acid (HF) and solvents namely ethylene glycol (EG), acetone, and ethanol were purchased from Sigma-Aldrich, Germany. EG-based electrolytes were used in combination with 0.2 M HF and 8 M water to grow TiO_2_ nanostructures. The used anodization conditions are listed in Table 1. All the anodization experiments were carried out at room temperature (∼20 °C) in a two-electrode system, using the Ti foil as the working electrode (anode) and a platinum gauze as the counter electrode. As-formed nanostructures were allowed to stand in ethanol for 2 h in order to remove organic components adsorbed from the electrolytic solution. This step was followed by washing of the nanostructures with distilled water and drying under nitrogen stream.

**Table 1:** Anodization conditions used for fabrication of TiO_2_ nanotubes

Diameter (nm)	Potential (V)	Anodization time (h)
15	10	2.5
50	20	2.5
100	58	2.5

### Characterisation of the Nanotube Arrays Morphology

The morphology of top view of the TiO_2_ nanostructures was observed using a field-emission scanning electron microscope (FE-SEM, Hitachi S4800). High contrast micrographs of nanostructured samples were obtained without any sputtering, as the obtained images already displayed reasonably good contrast.

The topographical features of Ti foil and TiO_2_-nanostructured surfaces were examined by Atomic Force Microscopy (Solver PRO, NT-MDT, Russia) in tapping mode in air. The samples were scanned with a standard Si cantilever at a constant force of 22 N/m and resonance frequency of 325 kHz (10-nm tip radius, 95-μm tip length). For comparison, the average surface roughness (*R*_a_) was calculated from 1 × 1μm^2^ area and the average values made on five different areas are presented.

### Characterisation of the Nanotube Arrays Surface Charge by Electrokinetic Measurements

The surface charge analysis of the pristine titanium foil and the NT arrays samples was carried out by an electrokinetic analyser (SurPASS, Anton Paar GmbH, Austria). The zeta potential (*ζ*) was derived by the measure of the streaming current (*I*_str_), according to the following equation [22]:

1 $$ \zeta \left({I}_{str}\right)=\frac{d{I}_{str}}{d\varDelta p}\cdot \frac{\eta }{\epsilon {\epsilon}_0}\cdot \frac{L}{A}, $$

where *dI*_str_/*d*∆*p* represents the streaming current dependence to the pressure difference ∆*p* between the ends of the capillary in the measuring cell, *η* is the viscosity, *ε* is relative permittivity, *ε*_0_ is the permittivity of the free space, and *L* and *A* are the length and the rectangular cross section of the capillary, respectively. According to the calibration, the precision of the instrument during the measurements was ±0.2 pH unit. Titration curves (dependence of *ζ* to the pH) were recorded in 0.001 mol/L biologically relevant media, in particular: sodium chloride saline solution (NaCl, Sigma-Aldrich), sodium chloride saline solution buffered with Tris(hydroxymethyl)aminomethane (TRIS, Alfa Aesar), phosphate buffer saline solution (PBS, tablets, Sigma-Aldrich), and Dulbecco’s modified Eagle medium (DMEM, GlutaMAX Supplement, Life Technologies). The influence of the electrolytic concentration on the *ζ* measurements was verified by systematically varying the molarity of the TRIS-buffered NaCl saline solution from 0.001 mol/L up to 0.1 mol/L.

## Results

Topographical investigations of the self-assembled TiO_2_ NT coatings were performed in terms of morphology and roughness prior any streaming potential measurement.

### Effects of the Synthesis Parameters on the Nanotubes Morphology

**Fig. 1:**
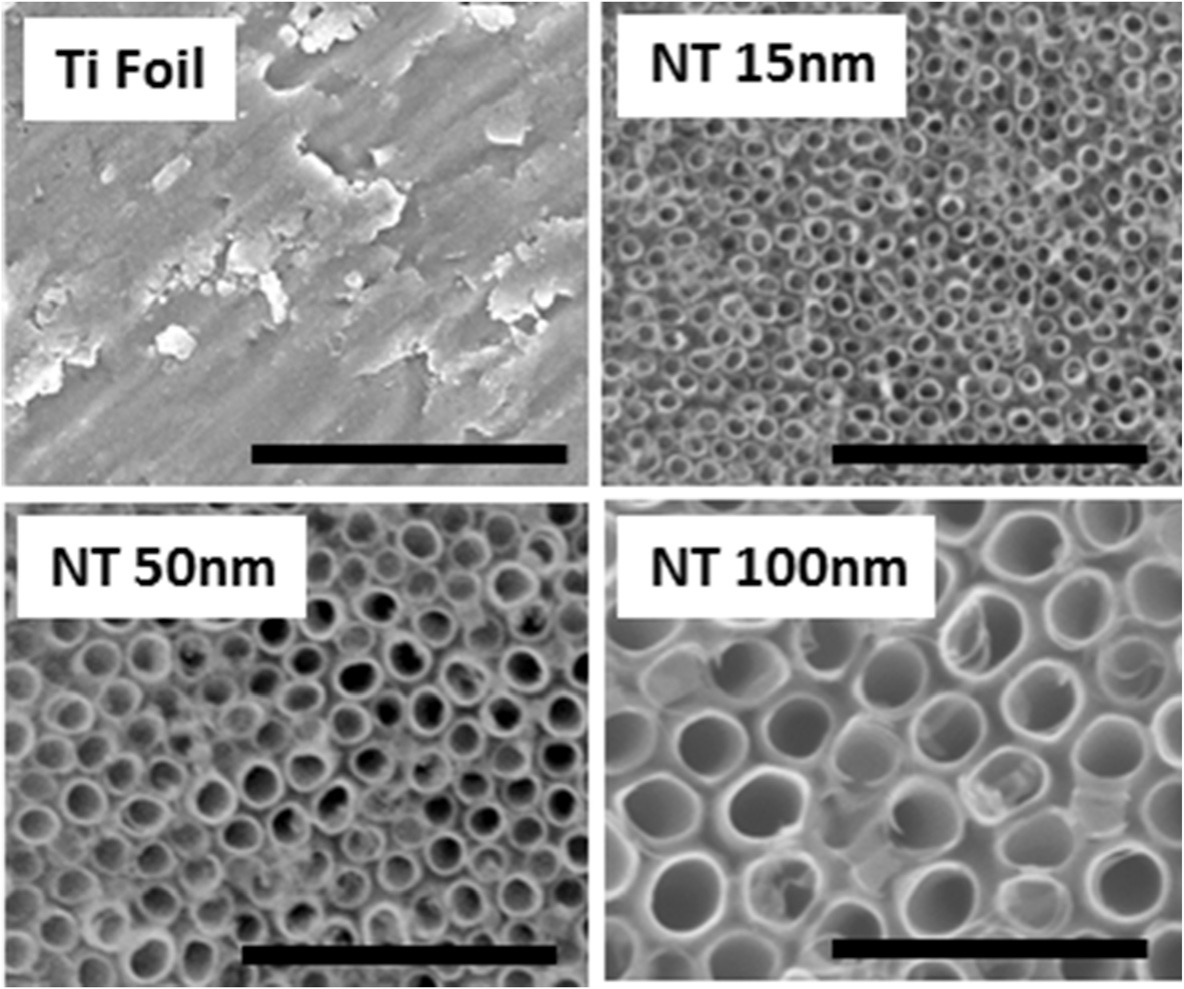
SEM micrographs (top view) of pristine Ti foil and TiO_2_ NTs with 15, 50, and 100 nm in diameter (*scale bar* 500 nm)

High-contrast SEM images of Ti foils and TiO_2_ nanostructures are presented in Fig. 1. From these images, it can be clearly seen that the diameter of nanotubes are 15, 50, and 100 nm, as expected by the choice of the synthesis parameters. SEM images also demonstrate the uniformity of nanotubes as expected, with standard deviations of 13.33, 10, and 5 % for nanotubes of 15, 50, and 100 nm in diameter, respectively. The standard deviation was calculated on nanotubes of all three diameters imaged by SEM at ×100,000 magnification.

**Fig. 2:**
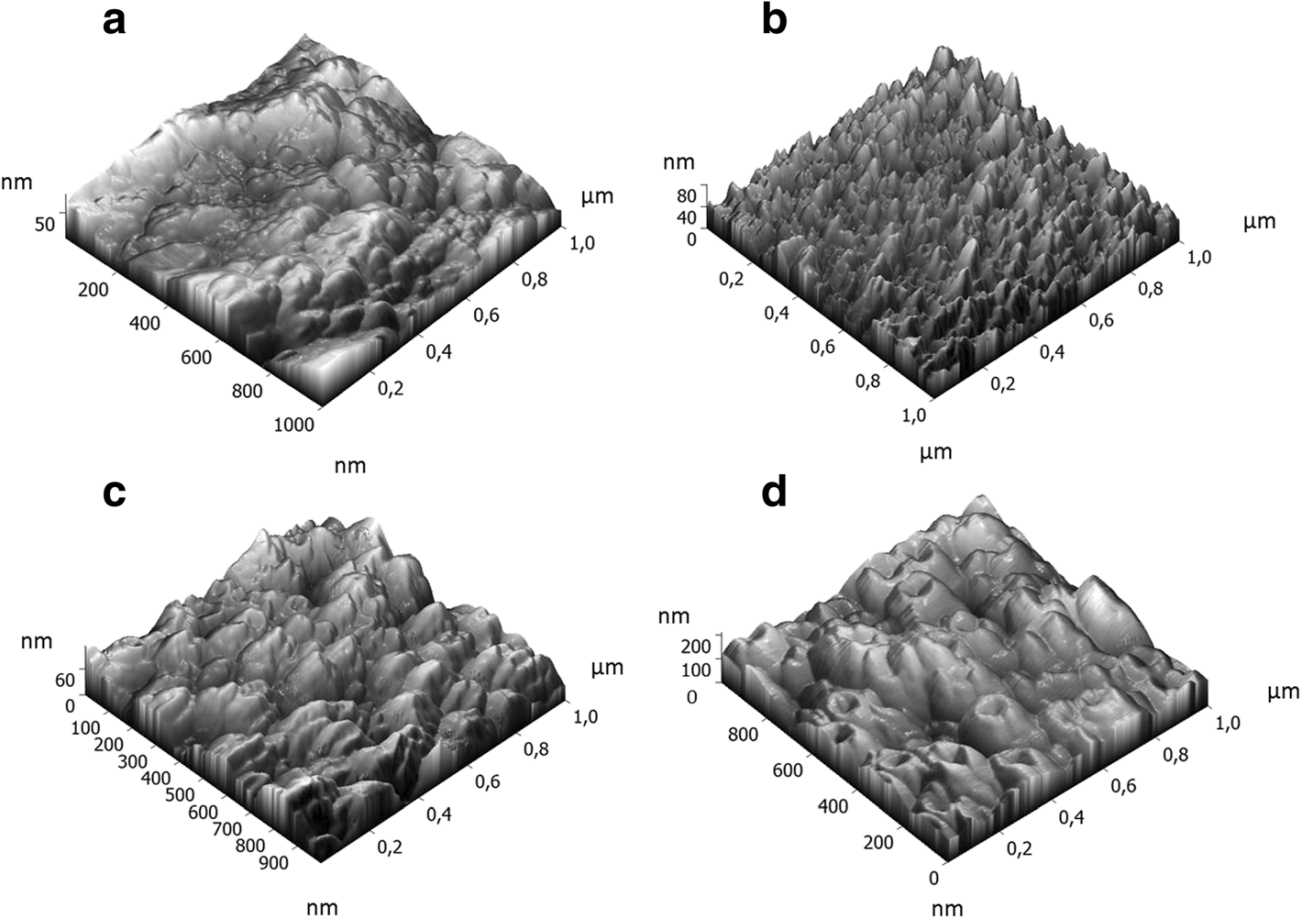
Topography of **a** pristine Ti foil and TiO_2_ NTs with **b** 15 nm, **c** 50 nm, and **d** 100 nm in diameter by AFM surface scan

Ti foil as well as nanotubes with 15, 50, and 100 nm in diameter was investigated by AFM to get more detailed information about the nanotopographic features (Figs. 1 and 2). From the AFM images, it is possible to observe changes in morphological features of the nanotubes with different diameters, with the NTs 15 nm diameter presenting the sharpest and highest amount of topographical spikes. Moreover, it can be noticed that already the Ti foil, which is used as a substrate for NTs growth, is not topographically uniform and some distinct features can be observed. The average surface roughness (*R*_a_) on the Ti foil was about 11.8 nm, while not much higher surface roughness was measured on NTs 15 nm and NTs 50 nm, with *R*_a_ about 9.1, and 15.5 nm, respectively. Conversely, the nanotubes with 100 nm in diameter presented an average surface roughness of about 25.5 nm. Nevertheless, these values are not fully representative of the surface topography, since the AFM tip is not able to penetrate within the whole length of the nanotube, so that it provides only information about the surface morphology, which further dictates surface interaction with the fluids.

### Effect of the Biological Media on the Measured *ζ* Values

In the first set of experiments, the electrolytic concentration was kept constant at 1.5 mM, while the nature of the present ions in solution varied.

**Fig. 3:**
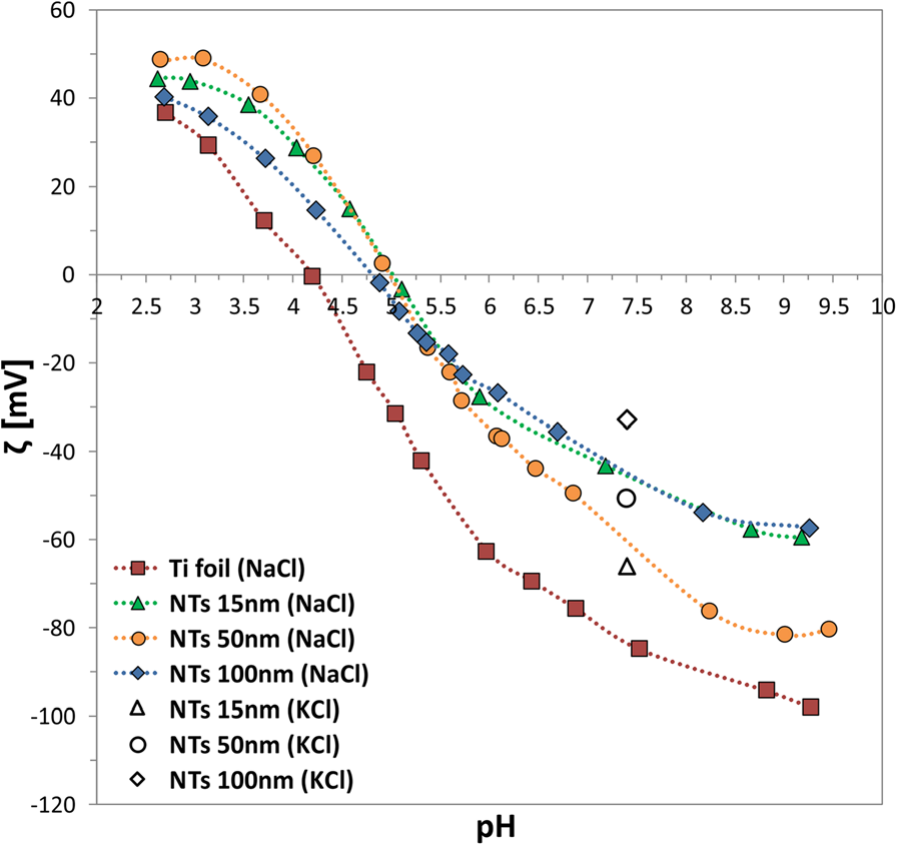
pH dependence of *ζ* for pristine Ti foil and TiO_2_ NTs with 15, 50, and 100 nm in diameter in 1.5 mM NaCl (in colours) and 1 mM KCl (between points at pH = 7.4, adapted from [16])

In 1:1 NaCl electrolyte, all the samples were negatively charged at physiological pH (pH = 7.4), but with different *ζ* magnitude, as shown in Fig. 3. Moreover, the pristine Ti foil presented a more acidic character (pH_IEP_ = 4.19) than the nanotube structures made of TiO_2_, which owned all very similar isoelectric points (IEPs), with pH_IEP_ varying from 4.81 to 5.01. For comparison, the *ζ* values at pH 7.4 previously obtained in 1 mM KCl (adapted from [16]) for each sample are also reported in Fig. 3 (between symbols). The curves belonging to NTs 50 and 100 nm in NaCl present a shift of around 15 mV towards more negative *ζ*-values in comparison to KCl; the NTs 15-nm curve represents an exception, with a shift towards less negative *ζ* (minor absolute values). Nevertheless, the IEPs in both NaCl (this study) and KCl [16] fall in the same pH region between ~4.6 and ~5.2. This is also in agreement with another study [23], where amorphous titania films were prepared by physical vapour deposition (amorphous titania films: pH_IEP_ ~4.6 in KCl). However, titanate nanowires measured by electrophoretic mobility resulted being a bit more acidic in KCl, with pH_IEP_ = 4.1 [24].

**Fig. 4:**
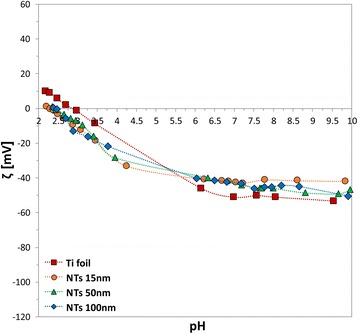
pH dependence of *ζ* for pristine Ti foil and TiO_2_ NTs with 15, 50, and 100 nm in diameter in 1 mM PBS

The IEPs shifted towards more acidic pHs for all the samples in PBS (Fig. 4) in comparison to the results in NaCl. Among them, the TiO_2_ NTs showed their IEP at pH ~2.4, while Ti foil at pH ~3. Looking at the titration curves, all the surfaces appeared negatively charged at physiological pH also in this case. However, the *ζ* magnitude shifted to lower (absolute) values, between −40 and −50 mV.

**Fig. 5:**
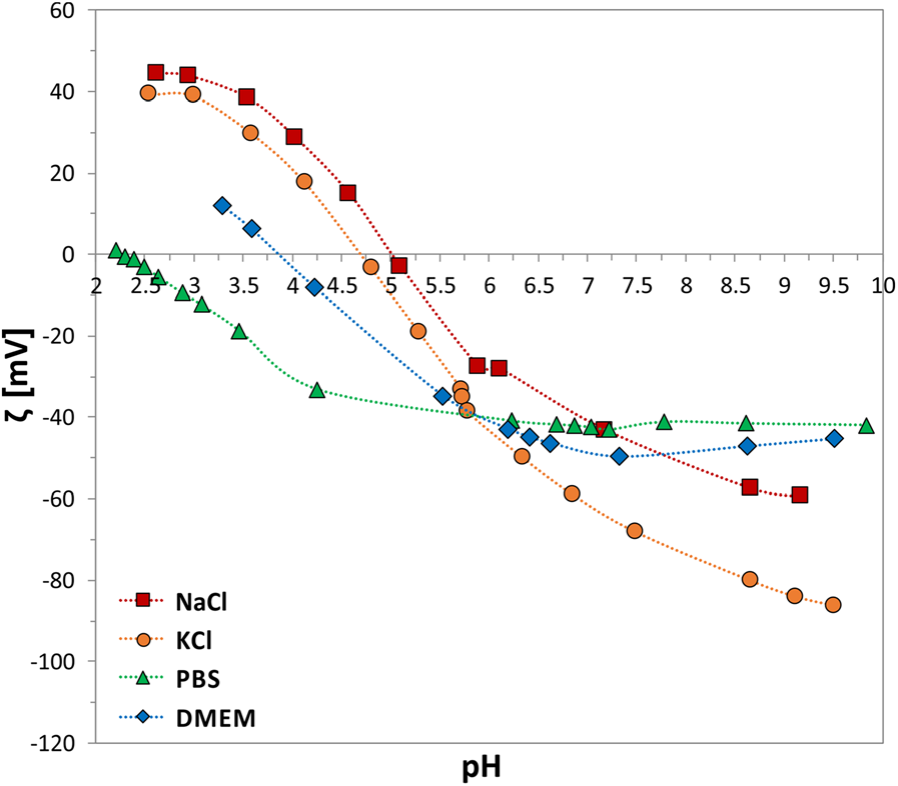
pH dependence of *ζ* for TiO_2_ NTs with 15 nm in diameter in NaCl, KCl (adapted from [16]), PBS, and DMEM

Figure 5 reports the titration curve of the self-assembled TiO_2_ nanotubes of 15 nm diameter (as representative sample) in DMEM cell medium, together with the titration curves in KCl [16], NaCl and PBS (for comparison). The IEP in 1 mM DMEM is at pH ~4, while the *ζ* at physiological pH is in the order of −50 mV.

### Effect of the Electrolytic Concentration on the *ζ* Values

The second set of experiments considered the influence of the electrolytic concentration on the Debye length and the width of the electrical double layer. NaCl was chosen as representative 1:1 electrolytic solution and the electrolytic concentration was systematically increased from 0.001 M up to 0.1 M (the limit of the electrokinetic analyser).

**Fig. 6:**
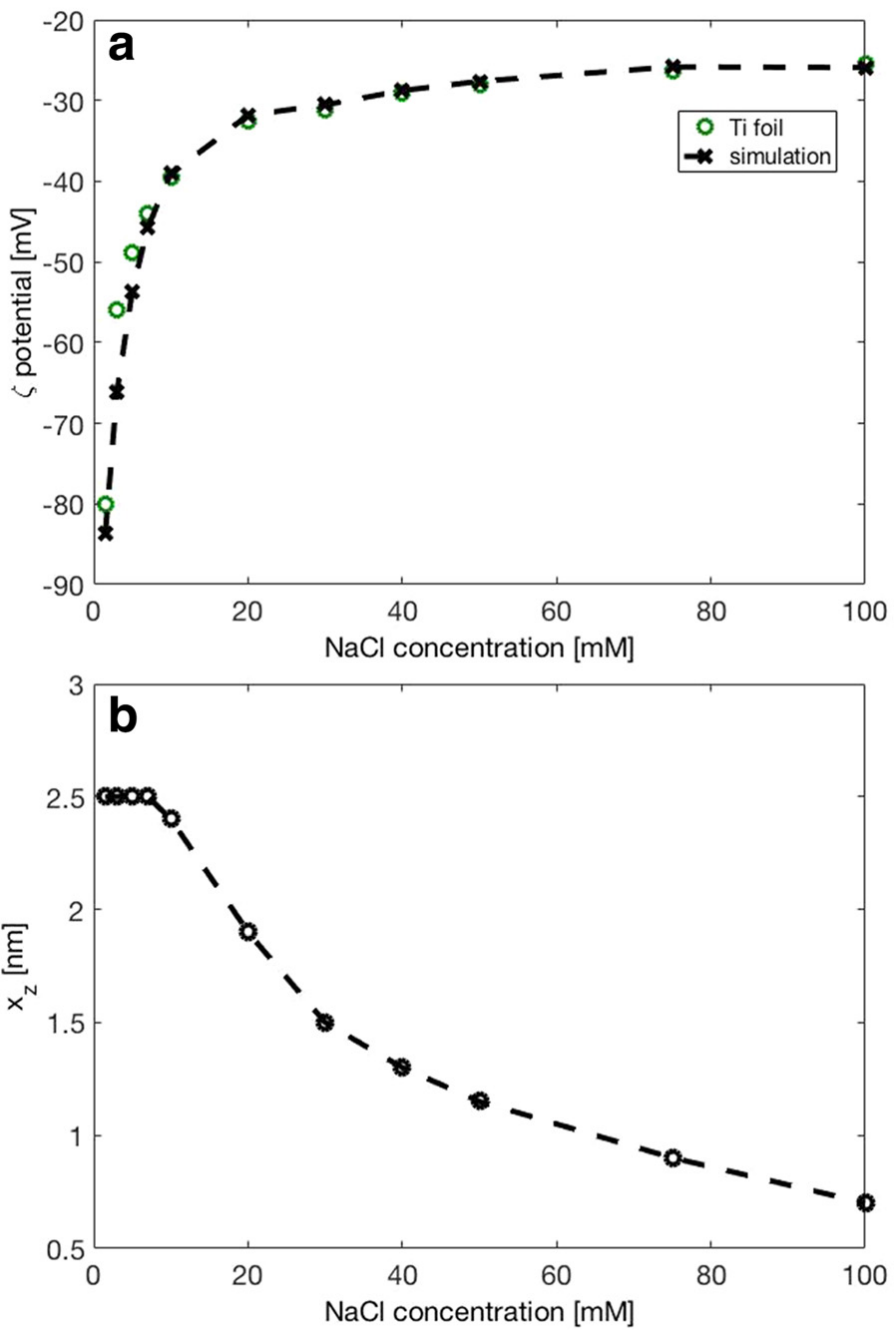
**a** Effect of the ionic strength of NaCl on zeta potential for flat Ti foil. Experimental values are denoted by *green circles*. The simulated data are denoted by the *dashed line* with *black crosses*. **b** Dependence of the distance *x*_z_ (position of the slip plane, see Fig. 7) on the NaCl concentration used in calculations presented in the upper panel. The parameters used in simulations are *σ* = −0.05 As/m^2^, *α*_-_ = 12, and *α*_+_ = 8. Parameters *α*_−_ and *α*_+_ describe the relative size of anions and cations, respectively (see Appendix)

**Fig. 7:**
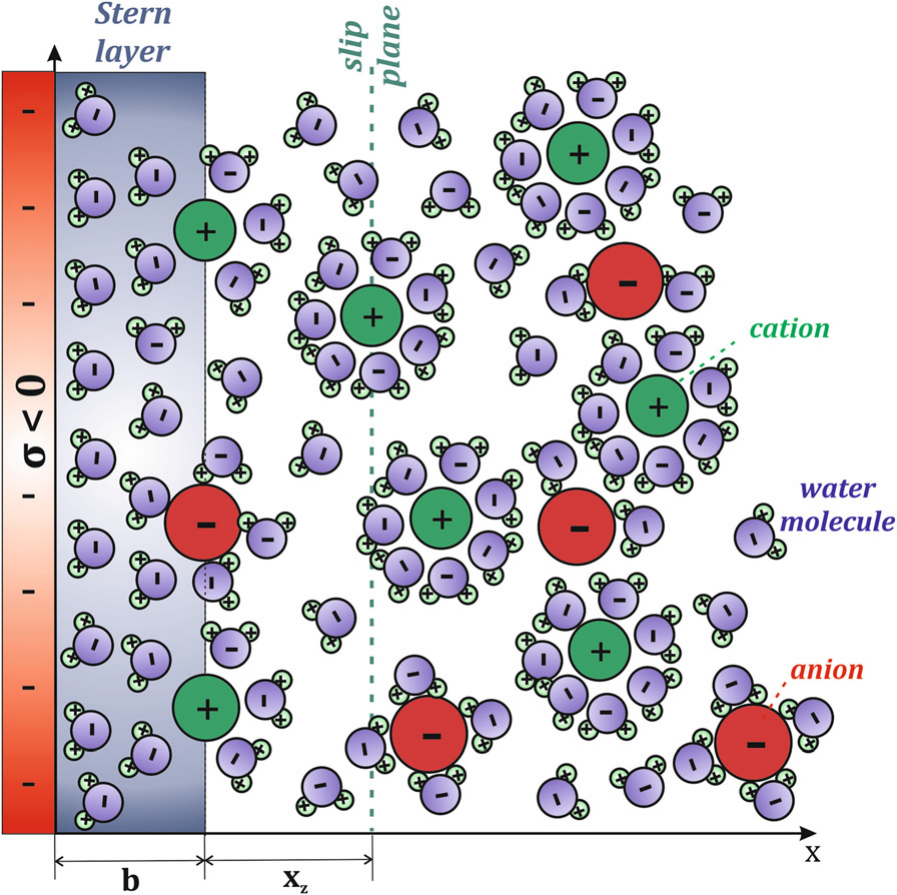
A schematic figure of the electric double layer showing an electrolyte solution (co-ions, counterions, and water molecules) in contact with a negatively charged planar Ti surface (*σ <* 0). The distance *b* denotes the distance of the closest approach (also called outer Helmholtz plane), approximately equal to the hydration radius of the counterions (cations in our case). The symbol *x*_*z*_ denotes the location of the fluid dynamic slip plane, where the electrolyte solution starts to move relative to the plane at *x* = 0 and where the zeta potential (*ζ*) is measured. The slip plane is usually beyond the thickness of Stern layer [36]

As shown in Fig. 6a, at constant pH (pH = 7.4), the measured magnitude of *ζ* for Ti foil had an exponential trend of decreasing value with decreasing NaCl concentration. The experimental dependence of *ζ* potential on NaCl concentration in Fig. 6a were analysed and interpreted by a theoretical mean-field model of electric double layer (Fig. 7), which takes into account the orientational ordering of water dipoles and the asymmetric finite size of the hydrated anions and cations in the electrolyte solution (see Appendix for details). The surface charge density of Ti foil *σ* = −0.05 As/m^2^ was determined by the comparison of experimental data and theoretical predictions in Fig. 6. As it can be seen in Fig. 6b, in the theoretical model, the position of the slip plane, determined by the distance *x*_z_ from the outer Helmholtz plane (OHP) (Fig. 7), should depend on NaCl concentration in order to get a better agreement between the experimental and theoretical points. The dependence of the position of the slip plane to the electrolyte concentration, which is usually beyond the thickness of Stern layer [12, 13, 16], is an expected result which follows from the comparison between experimental and theoretical values, predicted also by other authors [12, 13, 16]. The value of *x*_z_ = 2.5 nm at lower salt concentrations as determined in Fig. 6b was predicted also in [12, 13, 16].

**Fig. 8:**
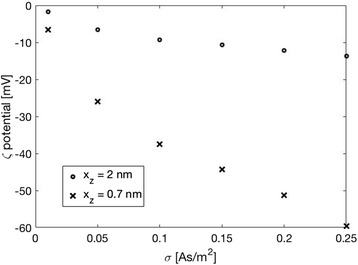
Calculated *ζ* potential as a function of surface charge density *σ* for two values of the position of the slip plane (defined by the distance *x*_z_ as shown in Fig. 7). The parameters used in the simulations are: NaCl = 100 mM, *σ* = −0.05 As/m^2^, *α*_-_ = 12, and *α*_+_ = 8

The value of the surface charge density *σ* = −0.05 As/m^2^ for Ti foil, which was estimated from the experimentally determined values of *ζ* potential (Fig. 6), depends on the selection of the values of the model parameter *x*_z_ at different salt concentrations. As illustrated also in Fig. 8, the surface charge density *σ*, *ζ* potential and the distance *x*_z_ are within our theoretical model of electric double layer interdependent quantities. Therefore, it was necessary to determine the surface charge density *σ* of Ti foil from the dependence of *ζ* potential on salt concentration in order to reduce the degree of freedom in determination of parameters *σ* and *x*_z_ while fitting the experimental dependence of *ζ* potential on NaCl concentration with theoretical curve as presented in Fig. 6a.

**Fig. 9:**
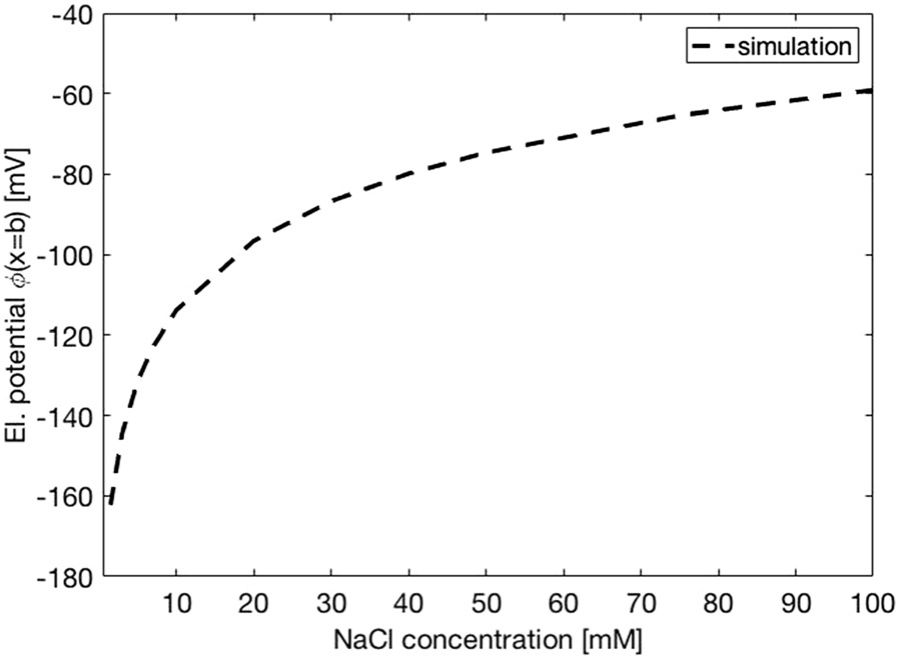
The effect of NaCl concentration on the electric potential value at *x* = *b* (i.e. at OHP). The parameters used in the simulations are *σ* = −0.05 As/m^2^, *α*_-_ = 12, and *α*_+_ = 8

It should be also stressed at this point that the electric potential at *x* = 0 (surface potential) and potential at *x* = b (potential at OHP) (see Fig. 7) are considerably more negative than *ζ* potential. To illustrate this difference, Fig. 9 shows the calculated electric potential at *x* = b (OHP) (see Fig. 7) for the same values of surface charge density *σ* = −0.05 As/m^2^ and same relative ions sizes *α*_−_ and *α*_+_ as used in Fig. 6. It can be seen that ф(*x* = b) is about twice more negative than *ζ* potential.

**Fig. 10:**
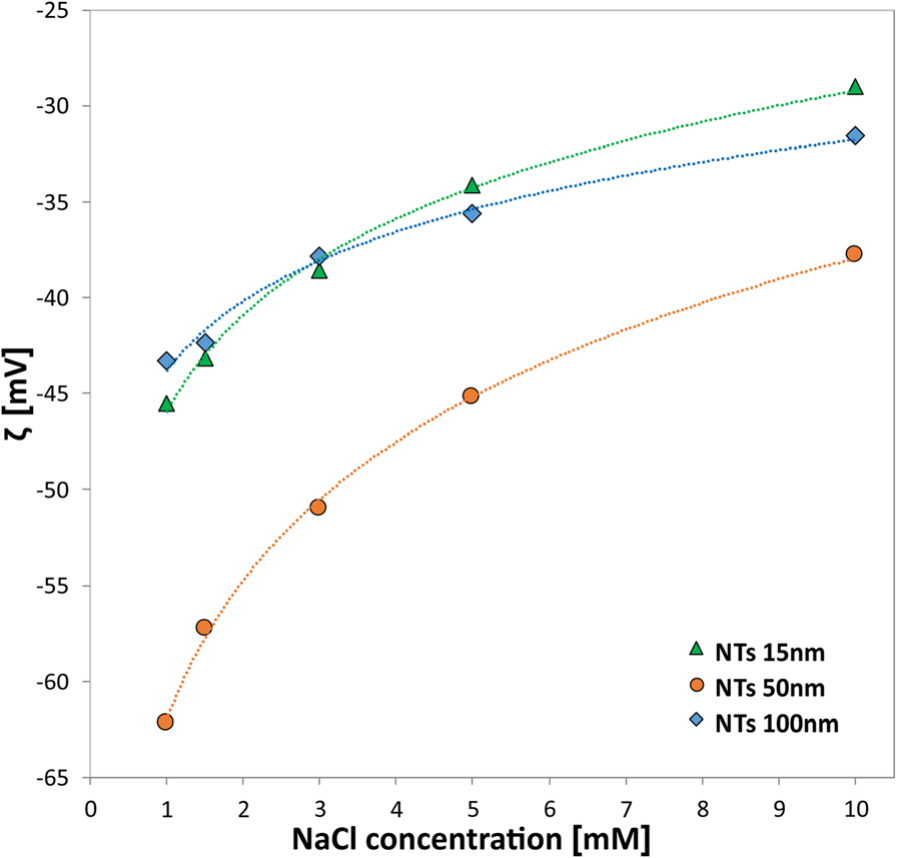
Effect of the NT diameter (15, 50, 100 nm) in relation to the increasing NaCl ionic strength from 1 to 10 mM (experimental data)

The similar decreasing exponential trend of *ζ*-values with decreasing NaCl concentration was observed also in the case of the self-assembled TiO_2_ vertically oriented NTs (Fig. 10). Since the drop of measured *ζ* magnitude is more prominent for small NaCl concentrations, Fig. 10 shows the dependence of *ζ* potential on NaCl concentration for different NT surfaces only for small values of NaCl concentrations, namely five electrolytic concentrations in the range between 1 and 10 Mm. The experimental values of *ζ* given in Fig. 10 are in agreement with the titration curves in Fig. 3. It can be also seen from Figs. 6a and 10 that the values of *ζ* for Ti foil are the most negative and considerably less negative for NTs 15 and 100 nm. The measured values of *ζ* for NTs 50 nm are surprisingly closer to the corresponding values of *ζ* potential for Ti foil than to the values of *ζ* for NTs 15 and 100 nm.

## Discussion

It is well known that the interaction and acceptance of a biomaterial at the biological level strongly depend on the surface properties of the implant itself. If on one hand the mechanical performances account on the bulk properties of the material, the surface properties such as charge, topography, roughness, and wettability are responsible for the first biological events after implantation. The growth of TiO_2_ nanotubes on Ti-based implants by electrochemical anodization has been reported to be a promising method to tailor the abovementioned surface properties at the nano- and micro-level, providing nano-roughness, high effective surface area and porosity, chemical stability, and high hydrophilicity [9, 10, 25]. However, due to technical limitations, not so much is known about the surface charge of TiO_2_ NTs-coated titanium implant surfaces. In our previous studies [12, 16], streaming potential technique was successfully applied to assess zeta potential values of titanium dioxide structures on Ti surfaces. In the case of NTs [16], the titration curves in very diluted, monovalent electrolytic solution (KCl) showed that all the surfaces were negatively charged at physiological pH, but they varied sequentially in *ζ* magnitude by changing the top diameter of the tubes [16]. Proceeding from these experiences, the current research concerned the systematic study of the surface charge properties in biologically relevant media of TiO_2_ NT arrays grown on titanium substrates, in dependence of their NTs diameter, the electrolytic concentration, and the type of media.

Tailoring the synthesis parameters during the electrochemical anodization resulted into nanotubes with controlled diameters (with variable porosities). SEM (Fig. 1) and AFM (Fig. 2) analyses confirmed about the uniformity of nanotubular surface and hollowness of TiO_2_ nanostructures. The experimental results show that by appropriate electrochemical anodization conditions, it is possible to form uniform layer of nanotubes with 15, 50, and 100 nm in diameter. Differences in the average surface roughness of nanotubes and Ti foil were also experimentally evaluated, and it was shown that the most prominent increase in surface roughness was observed for the 100-nm nanotubes. These surface features, together with the information about the zeta potential values measured for biologically relevant electrolytes, provide important information on the interfacial phenomena which could be transferred into relevant biological environment (body implants).

**Fig. 11:**
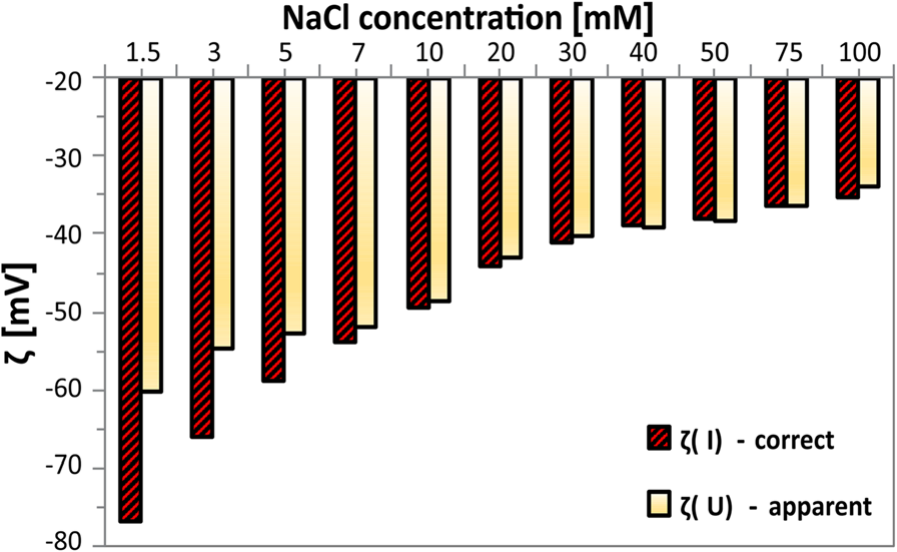
Zeta potential for Ti foil in NaCl at different concentrations. Data evaluated by streaming potential *ζ*(U) (apparent) vs. current potential *ζ*(I) (correct)

As described in the “Methods” section, in this work the values of zeta potential of the different samples were calculated from Eq. 1, i.e. evaluating the streaming current (*I*_str_), instead of the streaming potential (*U*_str_). This the only accurate approach to exclude the contribution to the conductance by materials which own an intrinsic, electrical conductance, such as metallic Ti foil or semiconductive TiO_2_ NTs. The enormous effect of the apparent zeta potential derived from the *U*_str_ is evident in Fig. 11. The effect is reduced at high salt concentrations, but never completely suppressed. Accordingly, all the zeta potential calculations were performed based on *I*_str_ by applying Eq. 1.

It has to be mentioned here that the variability of the samples in terms of nanotubes diameter distribution (see Fig. 1) gave a certain variability also in the obtained zeta potential values, despite the high instrumental accuracy. In particular, the standard deviation calculated from the SEM micrographs match perfectly with the one observed for the experimental *ζ*-values. Accordingly, the accuracy of the zeta potential measurement is higher than the homogeneity of the nanotubular structures.

The experimental quantification of surface charge for different TiO_2_ NT surfaces (NTs-coated Ti-foil) was assessed in various electrolytic solutions, relevant for the biological assessment. The electrolytic concentration was kept very low (1.5 mM) to render the measurements independent to the salt concentration (ionic strength). This allowed to observe the effect of monovalent, divalent, or multivalent ions in the electrolytic solution on the compression of the electrical double layer.

Firstly, NaCl saline solution, widely used for its isotonicity with blood and tissues, was chosen as 1:1 physiological electrolyte (Fig. 3). All the self-assembled TiO_2_ NT arrays owned very similar IEPs (pH ~5), despites the different top NT diameters (15, 50, and 100 nm). However, all the samples were negatively charged at physiological pH, as already observed in the case of KCl 1:1 electrolyte [16], but they differed in magnitude. Nevertheless, the lowest recorded *ζ* value among the samples at pH = 7.4 was around −50 mV, which would correspond to nicely dispersed particles in case of suspensions. Therefore, it can be presumed that the analysed surfaces might display a little higher surface charge density, as estimated in the analysis presented in Fig. 6.

Nevertheless, some interesting differences can be noticed if the results in NaCl and in KCl [16] at physiological pH are compared. Even though both these salts give rise to 1:1 electrolytes, Na^+^ and K^+^ cations differ in specificity to the surface and in their hydrated ionic radius. The ionic specificity gives rise to a specific adsorption on the surface [26]. At pH greater than the IEP, the ionic affinity for negatively charged, hydrophilic surfaces (TiO_2_ nanotubular surfaces in this work) is expected to follow the indirect Hofmeister series [27]. Accordingly, Na^+^ would better stabilise the electrical double layer than K^+^, giving higher *ζ* in magnitude (this study) and i.e. reducing the agglomeration in case of colloidal particles [27]. The Hofmeister series obey also the hydration rate of ions.

**Fig. 12:**
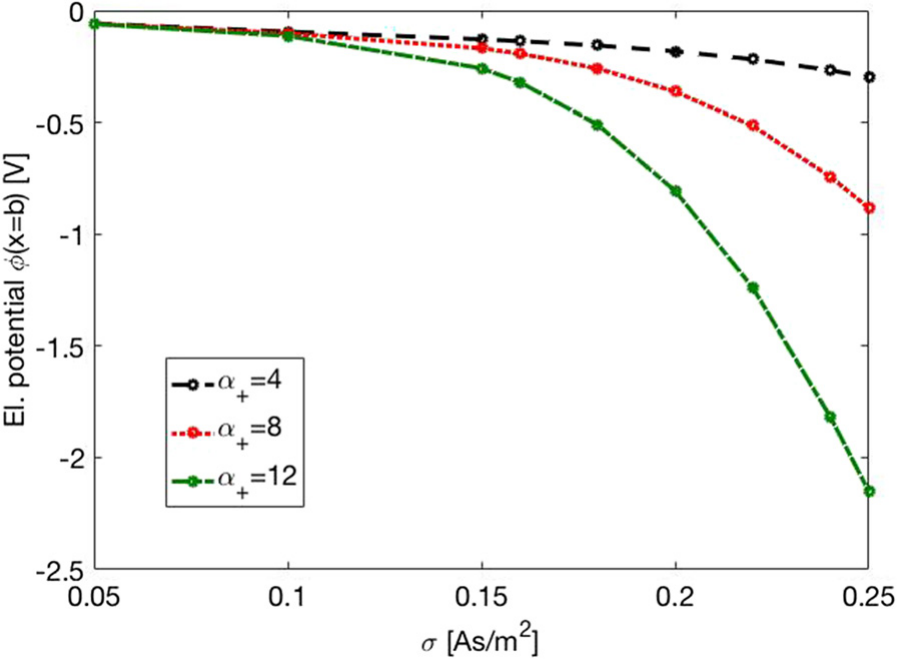
Zeta potential at *x* = *b* (i.e. at the outer Helmhotz plane, see also Fig. 7) as a function of surface charge density of a flat surface (*σ*) calculated for three values of parameter *α*_+_ describing the size of cations (larger *α*_+_ corresponds to larger size of hydrated cation, see also Appendix). The values of other model parameters used in the simulations are: NaCl concentration = 100 mM and *α*_-_ = 12

The hydrated ionic radius of the cation (counterions) determine its maximum possible concentration at the charged surface; therefore, it has an influence on the diffuse double layer spatial distribution [28]. In agreement with [22, 28–30], larger counterions produce a more extended double layer and, consequently, a more negative the zeta potential, as shown by the calculated theoretical values presented in Fig. 12. As it can be seen in Fig. 12, the influence of the counterion size on the value of zeta potential at OHP becomes very strong for large magnitudes of the surface charge density *σ*. Oppositely, at small magnitudes of the surface charge density *σ*, the influence of counterion size on the electric potential is much weaker and it varies in the order of magnitude of 5 mV.

The experimentally determined difference in zeta potential of Ti foil between NaCl and KCl electrolyte is around 5–10 mV, which indicates that we have probably selected too small magnitude of surface charge density *σ* in the theoretical description of the experimental results for Ti foil presented in Fig. 6. Hence, *σ* value of Ti foil might be actually more negative than the estimated value −0.05 As/m^2^.

As shown by the theoretical calculations (Fig. 12), larger cations produce more negative surface potential at OHP than a smaller cation. Since hydrated Na^+^ are larger than K^+^, the theoretically predicted values of *ζ* potential for Ti foil and/or TiO_2_ NT surfaces are expected to be more negative in NaCl than in KCl, for about 10 mV (see Fig. 12 and [28]). This behaviour complied with our experimental results for the NTs 50 nm (Fig. 3, orange circles) and NTs 100 nm (Fig. 3, blue rhomboids), while NTs 15 nm acted as an exception (Fig. 3, green triangles), since the experimental data reveal lower absolute *ζ*-values (less negative charge) in NaCl than in KCl. This could be ascribed to several contingent factors, besides the variability among the samples, which inevitably occurs during the synthesis. For instance, NTs 15 nm presents very specific surface characteristics in comparison to the other two types of NT samples. In fact, NTs 15 nm owns a higher average surface roughness (Fig. 2), a higher surface porosity (Fig. 1), a higher total length of sharp edges of NT walls per unit top surface area (see also [9, 10, 25]), and the highest (accessible) surface area. All these features of NTs 15 nm may generate an “internal” streaming current within the hollow interiors of NTs 15 nm and also vortexes at the NTs surface, in addition to the “external” streaming current in the measuring capillary. Due to particular behaviour, the *ζ* computed by the Smoluchowski method (Eq. 1) results as “apparent” and rated too low/different. In addition, due to vortexes (turbulent flow), the basic assumption on which the Smoluchowski method was originally derived, i.e. the assumption of laminar flows, is violated. Accordingly, gap height dependence measurements [13] would be needed to assess more realistic *ζ* potential values (proposed as our next step).

Also, the wettability and reactivity of the hydroxyl groups on the surface “as-prepared” and “aged” have to be taken into account while interpreting the presented experimental results. Namely, already after a few hours from the preparation, the contact angles of the self-assembled nanotubes structures rise from super-hydrophilic (as-prepared) to hydrophobic by collecting organic contaminants (hydrocarbons) from the environment, if not protected in a controlled atmosphere or treated i.e. with gaseous plasma [31]. NTs 15 nm sample shows the least hydrophilic surface among the three types of NT diameters, indicating a minor amount of free hydroxyl groups and less surface acidity, contributing to inferior *ζ*-values in magnitude.

All the differences among samples detected in 1:1 electrolytes were then mostly suppressed when multivalent ions electrolytes were used for the studies. Phosphate saline buffer solution was chosen as 1:3 electrolyte, containing monovalent cations such as Na^+^ and K^+^, and trivalent phosphate anions PO_4_^3−^ (Fig. 4). In comparison to the NaCl case, a noteworthy shift of the IEP towards acidic pH was observed for all the surfaces in PBS, with a more prominent effect for the tubular nanostructures. This indicates the occurrence of a preferential adsorption of phosphate anions on the top edges of the TiO_2_ NTs rather than on Ti foil, as previously indicated in [12] for TiO_2_ nanocrystalline films, even though no significant discrepancy was observed among the different NT diameters. In terms of magnitude of *ζ* potential, all the curves shifted and converged in a range between −40 and −50 mV at physiological pH. This behaviour may be ascribed to the effect of the multivalent phosphate ions (see also [9, 10, 25]). In fact, at constant electrolytic concentration (very low in our case 0.001 mM), the higher is the electrolyte valence, the larger is the ionic strength. In turn, since the Debye length is inversely proportional to the square root of the ion ionic strength, it is straightforward that the electric double layer shrank in presence of multivalent electrolytes (due to their strong screening effect) and, consequently, the surface charge appeared reduced.

In our experiments, when the electrolytic solution was exchanged with DMEM cell medium (to better mimic the real composition of body fluids), the TiO_2_ nanostructured surfaces experienced a completely different environment than in the previously described cases. DMEM solution contains very diverse ions, ranging from the monovalent ones (Na^+^, K^+^, Cl^−^), the divalent ones (Ca^2+^, Mg^2+^), to the multivalent ones (PO4_4_^3-^, SO_4_^2−^), as well as zwitterionic substances such as the amino acids. Such a variety of cations and anions gave rise to a competition not only among these counterions in solution to “neutralise” the surface charge of the scaffolds, but also among each other within the double layer, due to the reciprocal direct attraction/repulsion forces. Therefore, the results on the IEP and on the *ζ* magnitude (blue titration curve with rhomboids in Fig. 5) depend on the reached equilibrium between the electrostatic forces among all the species involved. Contrarily to the PBS case, where the multivalent ions were represented only by phosphates, in DMEM the mutual effect of multivalent cations and anions resulted in: (a) specific adsorption of phosphates and sulphates which rendered the surface more acidic (pH_IEP DMEM_ ~4) than in the case of NaCl (pH_IEP NaCl_ ~5) but less acidic than in PBS (pH_IEP PBS_ ~2.4); (b) no drastic effect on the double layer compression, with *ζ* values comparable with the ones in PBS.

As mentioned before, the first part of the results concerned the effect of ion valence on the extension of the electrical double layer. Thus, in some of the above-described experiments the electrolytic concentration was purposely kept very low, in order to exclude its effect on the ionic strength. However, in normal conditions, the biological fluids have a concentration of 0.15 M. Therefore, the influence of the solution concentration was also examined, by systematically varying the experimental electrolytic concentration till 0.1 M (Fig. 11). In order to avoid any impact of the valence, this time the 1:1 NaCl electrolyte was chosen.

**Fig. 13:**
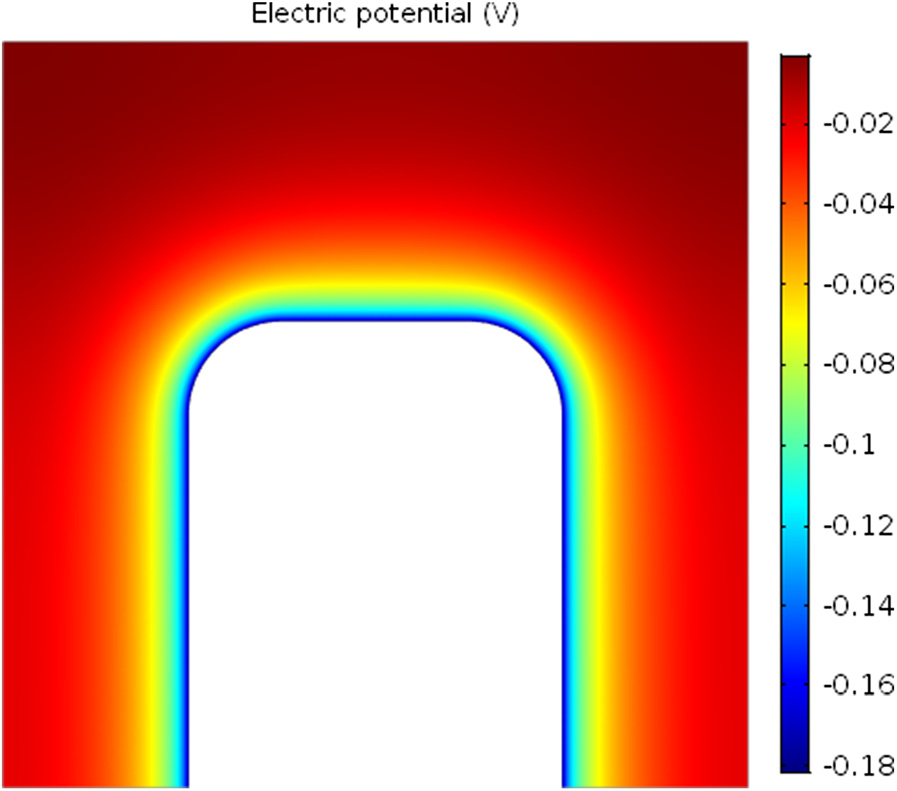
Calculated distribution of electric potential around the wall of TiO_2_ nanotube (in Volts). The white surface shows the cross section of the nanotube wall. The parameters used in the simulations are *σ* = −0.2 As/m^2^, NaCl concentration = 100 mM, *α*_−_ = 12 and *α*_+_ = 8

**Fig. 14:**
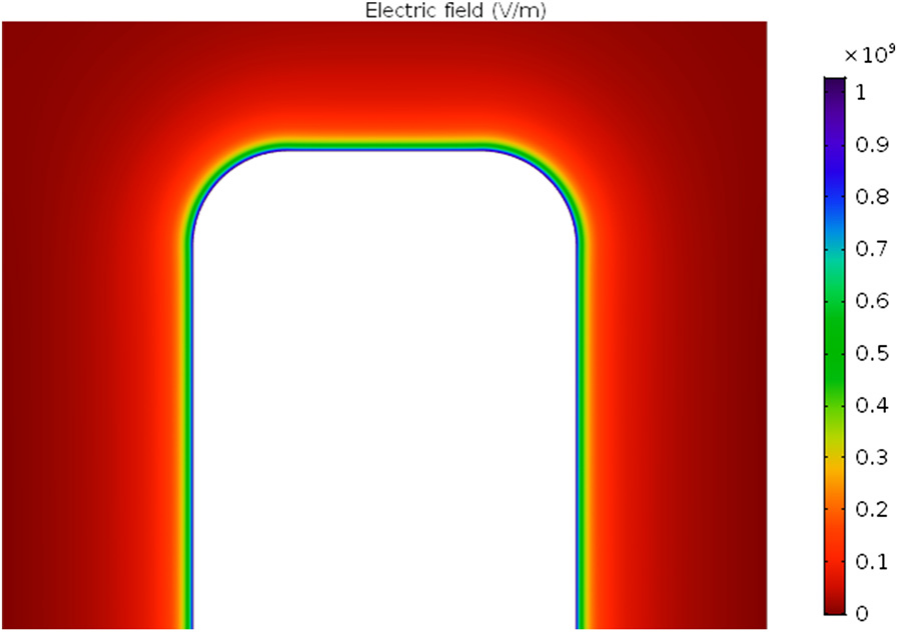
Calculated electric field magnitude around the wall of TiO_2_ nanotube (in V/m). The white surface shows the cross section of the nanotube wall. The parameters used in the simulations are *σ* = −0.2 As/m^2^, NaCl concentration = 100 mM, *α*_-_ = 12 and *α*_+_ = 8

**Fig. 15:**
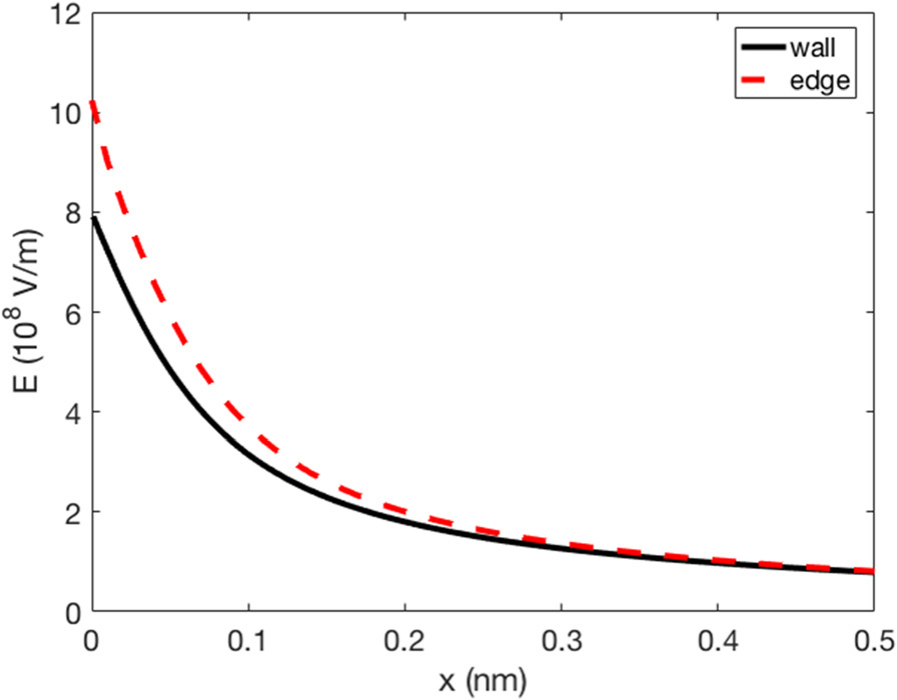
Calculated magnitude of the electric field strength as a function of the distance from the surface in normal direction to the surface (*d*) in the case of NT wall surface far from the top edges (*full line*), and at the highly curved top edge region (*dashed line*). The parameters used in the simulations are *σ* = −0.2 As/m^2^, NaCl concentration = 100 mM, *α*_−_ = 12 and *α*_+_ = 8

The complementary theoretical analysis of the experimentally observed *ζ* potential dependence on the NaCl concentration was performed within GI electric double layer model [22]. As shown in Figs. 6 and 9, the calculated *ζ* potential decreases in magnitude with the increase of electrolyte concentration. This means that at higher salt concentrations the electrostatic repulsion becomes weaker due to the vicinity of the ions, causing a shrink of the electrical double layer thickness [9, 10, 25]. The results presented in the above mentioned Figs. 6 and 9 consider the simplest case of flat nonporous titanium foil with homogeneous distribution of electric charges on its surface, i.e. constant surface charge density *σ* over the entire surface. On the other hand, theoretical consideration of porous TiO_2_ surfaces, as TiO_2_ NT surfaces, would demand more complex numerical modelling to capture the complicated geometry of the nanotubular surface. The modified GI theory can be applied to describe various nanotubular surfaces, including those of different nanotubular diameter. The development of a realistic 3-dimensional FEM geometrical model appears to be the main problem. However, this issue can be numerically solved with high enough accuracy and without spending too much computer time. The calculated space distribution of the electric potential (Fig. 13) and electric field strength (Fig. 14) strongly vary in the vicinity of the nanotube wall surface, and is therefore much different on the surface of NTs in comparison to the values in the hollow interior of NTs. This fact leads us to the conclusion that the measured values of *ζ* potential should be considered as effective macroscopic parameters which only roughly describe the electrical properties of TiO_2_ nanotubular surfaces with different diameters. Our preliminary numerical results also show that the electric potential and electric field strength on the NT surface only slightly depend on the diameter of NTs. In agreement with [15], Fig. 15 show that the magnitude of electric field strength is increased at highly curved edge of the NT wall top surface. This means that the main parameter determining the value of *ζ* potential of NT surfaces is the total length of NT top edges per unit area of the NT surface.

In the experiments, the decrease of *ζ* with the increase of the electrolyte concentration, observed for flat Ti foil, is preserved also for 15, 50, and 100-nm NT-coated surfaces (Fig. 10), where the effective/average surface charge density *σ* obviously depends on the NT diameter and surface roughness. Based on the experimental results presented in Fig. 10, it can be concluded that among the chosen NTs-coated surfaces (Figs. 1 and 2), the TiO_2_ surfaces formed by NTs with 100-nm diameter display the lowest exposed top surface area (due to the most voids) and the lowest effective/average surface charge density *σ* among the three NT diameters. In contrast, the substrates covered by NTs with 15 nm diameter present the highest exposed surface area and the highest effective/average surface charge density, partially ascribable to the longest total length of sharp convex top edges of NT walls [15]. The major influence of NT voids on the magnitude of *ζ* is expected for the NTs 100 nm diameter, while a weaker effect is expected for the NTs 50 nm diameter, and even less for the NTs 15 nm diameter. The assumed trend is actually obeyed in experiments only in the case of NTs 50 nm and NTs 100 nm, while NTs 15 nm showed a distinctive behaviour, owning experimentally determined value of *ζ* magnitude very similar to the one of NTs 100 nm at all the considered NaCl concentrations (Fig. 10). As in the case of the titration curves in NaCl (Fig. 3), diverse phenomena like for example surface roughness, not included into the experimental determination of *ζ*(*I*_str_), can anyway influence the obtained data for NTs 15 nm and, therefore, partially explain the reason of deviation from the expected results for NTs 15 nm.

## Conclusions

This study presents results on surface charge measurements of electrochemically anodized TiO_2_ nanotubes, proposed as materials for body implants, in biologically relevant electrolytes (NaCl, PBS, cell medium). The use of an electrokinetic analyser allowed for the systematic accomplishment of zeta potential titration curves at low electrolytic concentration (10^−3^ M), single points at fixed—physiological—pH and at various electrolytic concentrations (up to 0.1 M). Accordingly, the results were presented considering the effect of the ionic strength, as well as the multivalence of the electrolytes in solution. Zeta potential was shown to be mostly influenced by the ionic strength, while the IEPs were mostly affected by the valence and charge of the electrolytic ions, as a proof of their adsorption/interaction with the surfaces. Also, the effects of surface porosity and the surface area, exposed to the solution, were taken into account. Hence, several hypotheses were formulated to explain the behaviour of the three different morphological dimensions of NTs (with 15, 50 and 100 nm in diameter), i.e. considering their topographical characteristics, as well as their wettability and the ion affinity towards the surfaces. The experimental data were supported by the theoretical model, adjusted to our specific system/cases, but applicable also to other nanoporous structures.

Overall, the outcomes definitely broad the scenario of the characterisation of the implant surfaces at the bio-interface. However, in the current study the effect of surface porosity and roughness to the conductance and “apparent” streaming current effects have not been quantitatively assessed; therefore, gap height dependence measurements have been planned as the next step of our investigations.

## Appendix

### Calculations on the Spatial Dependence of the Electric Potential

The spatial dependence of electric potential in electrolyte solution in contact with negatively charged flat Ti surface is calculated according to the modified GI model [32], as a generalisation of Wicke and Eigen model [33, 34] and GI model [35]. The modified GI model [35] simultaneously takes into account the asymmetry of the anion and cation finite sizes and the spatial dependence of the polarisation and relative permittivity due to the orientational ordering of water molecules close to the charged surface (Fig. 7).

The expressions for the spatial distribution of cations *n*_+_(*x*)), anions *n*_−_(*x*)), and water dipoles *n*_*w*_(*x*)) in the electric double layer within modified GI model are [32]:

A1 $$ {n}_{+}(x)={n}_0{e}^{-{e}_0\phi \beta}\frac{n_s}{\mathcal{D}\left(\phi,\ E\right)}, $$

A2 $$ {n}_{-}(x)={n}_0{e}^{e_0\phi \beta}\frac{n_s}{\mathcal{D}\left(\phi, E\right)}, $$

A3 $$ {n}_w(x)=\frac{n_{0w}{n}_s}{\mathcal{D}\left(\phi, \kern0.5em E\right)}\frac{ \sinh \left(\gamma {p}_0E\beta \right)}{\gamma {p}_0E\beta }, $$

which then appear in the corresponding Poisson’s equation:

A4 $$ \frac{d}{dx}\left[{\varepsilon}_0{\varepsilon}_r(x)\frac{d\phi }{dx}\right]=2{e}_0{n}_s{n}_0\frac{ \sinh \left({e}_0\phi \beta \right)}{\mathcal{D}\left(\phi,\ E\right)}. $$

The spatial dependence of the relative permittivity *ε*_r_(*x*) is:

A5 $$ {\varepsilon}_r(x)={n}^2+{n}_{0w}{n}_s\frac{p_0}{\varepsilon_0}\left(\frac{2+{n}^2}{3}\right)\left(\frac{\mathrm{\mathcal{F}}\left(\gamma {p}_0E\beta \right)}{\mathcal{D}\left(\phi,\ E\right)E}\right) $$

and

A6 $$ D\left(\phi, E\right)={\alpha}_{-}{n}_0{e}^{e_0\phi \beta }+{\alpha}_{+}{n}_0{e}^{-{e}_0\phi \beta }+\frac{n_{0w}}{\gamma {p}_0E\beta } \sinh \left(\gamma {p}_0E\beta \right), $$

where the function ℱ(*u*) is defined as ℱ(*u*) = ℒ(*u*)(sinh *u*/*u*). Here *n*_0*w*_ is the bulk number density of water molecules, *n*_0_ is the bulk number density of anions and cations, *n*_*s*_ is the number density of lattice sites, *β* = 1/kT, *k* is the Boltzmann constant, *T* is the absolute temperature, *e*_0_ is the unit charge, *ϕ* is the electric potential, *p*_0_ = 3.1, *D* is the magnitude of the external water dipole moment, *E* is the magnitude of electric field strength, *ε*_0_ is the permittivity of free space, and *n* 0 1.33 is the optical refractive index of water and γ is given by [32, 35]:

A7 $$ \gamma =\left(2+{n}^2\right)/2. $$

Further, *α*_+_ and *α*_*−*_ are the number of the lattice sites occupied by positive and negative ions, respectively, with:

A8 $$ {n}_s={\upalpha}_{+}{n}_{+}(x)+{\upalpha}_{-}{n}_{-}(x)+{n}_w(x). $$

The water (solvent) molecule is assumed to occupy one lattice site, where *n*_0w_/*N*_A_ = 55 mol/l.

In the bulk,

A9 $$ {n}_s={\upalpha}_{+}{n}_0+{\upalpha}_{-}{n}_0+{n}_{0w} $$

The boundary condition at the charged plate, i.e. at *x = 0* is:

A10 $$ {\left.\frac{d\phi }{dx}\right|}_{x=0}=-\frac{\sigma }{\varepsilon_0{\varepsilon}_s} $$

where *ε*_*s*_ is the relative permittivity in Stern layer. We assumed that there are no ions only water molecules in the Stern layer. Therefore, the relative permittivity in Stern layer (*x* ≤ 0 ≤ *b*) is:

A11 $$ {\varepsilon}_s={n}^2+{n}_s\frac{p_0}{\varepsilon_0}\left(\frac{2+{n}^2}{3}\right)\left(\frac{\mathrm{\mathcal{L}}\left(\gamma {p}_0E\beta \right)}{E}\right) $$

where *E* is constant in the Stern layer.

The condition at the boundary Stern layer, i.e. at *x = b* is:

A12 $$ {\left.{\varepsilon}_s\frac{d\phi }{dx}\right|}_{x={b}_{-}}={\varepsilon}_r{\left.\frac{d\phi }{dx}\right|}_{x={b}_{+}} $$

Since *dϕ*/*dx* in Stern layer is constant, it follows from Equations A11 and A12:

A13 $$ {\left.\frac{d\phi }{dx}\right|}_{x={b}_{+}}=-\frac{\sigma }{\varepsilon_0{\varepsilon}_r} $$

when *ε*_*r*_*(x)* is for *x > b* defined by Equation A5.
